# Assessment of Growth Using Mandibular Canine Calcification Stages and Its Correlation with Modified MP3 Stages

**DOI:** 10.5005/jp-journals-10005-1050

**Published:** 2010-04-15

**Authors:** Reshma Nayak, US Krishna Nayak, Gautam Hegde

**Affiliations:** 1 Postgraduate Student, Department of Orthodontics and Dentofacial Orthopedics, AB Shetty Memorial Institute of Dental Sciences Mangalore, Karnataka, India; 2 Dean Academics and Head, Department of Orthodontics and Dentofacial Orthopedics, AB Shetty Memorial Institute of Dental Sciences, Mangalore, Karnataka, India; 3 Former Postgraduate Student, Department of Orthodontics and Dentofacial Orthopedics, AB Shetty Memorial Institute of Dental Sciences, Mangalore, Karnataka, India

**Keywords:** MP3, mandibular canine calcification, growth prediction, skeletal maturity, modified MP3.

## Abstract

**Background and objectives:**

Orthodontic diagnosis and treatment planning for growing children must involve growth prediction, especially in the treatment of skeletal problems. Studies have shown that a strong association exists between skeletal maturity and dental calcification stages.

The present study was therefore taken up to provide a simple and practical method for assessing skeletal maturity using a dental periapical film and standard dental X-ray machine, to compare the developmental stages of the mandibular canine with that of developmental stages of modified MP3 and to find out if any correlation exists, to determine if the developmental stages of the mandibular canine alone can be used as a reliable indicator for assessment of skeletal maturity.

**Methods:**

A total of 160 periapical radiographs (80 males and 80 females), of the mandibular right canine and the MP3 region was taken and assessed according to the Dermirjian’s stages of dental calcification and the modified MP3 stages.

**Results:**

The correlation between the developmental stages of MP3 and the mandibular right canine in male and female groups, is of high statistical significance (p = 0.001). The correlation coefficient between MP3 stages and developmental stages of mandibular canine and chronological age in male and females was found to be not significant.

**Conclusions:**

The correlation between the mandibular canine calcification stages and MP3 stages was found to be significant. The developmental stages of the mandibular canine could be used very reliably as a sole indicator for assessment of skeletal maturity.

## INTRODUCTION

In preventive and interceptive orthodontics, it is required to know not only the exact chronological, dental or skeletal age of the patient, but also if the patient will grow during the treatment period and what percentage of growth can be expected during that time.^1^ The disadvantages of the routine methods of skeletal maturity were that, they required elaborate equipments, were expensive and the radiation exposure time and dose were high ^2^ Studies by on American population have shown that relationships between the stages of tooth mineralization of the mandibular canine appear to correlate better with skeletal maturity indicators than the other teeth.^3,4^

The present study was, therefore taken up to provide a simple and practical method for assessing skeletal maturity using a dental periapical radiographic film and a standard dental X-ray machine, to corelate the developmental stages of the mandibular canine with that of known method like skeletal developmental stages of MP3^2,5^ and to find out if the developmental stages of the mandibular canine alone can be used as a reliable indicator for assessment of skeletal maturity.

## METHODOLOGY

The study was conducted on 160 South Indian subjects (80 males and 80 females) between 8 to 16 years of age (Table 1).

**Table 1:** **Table 1:** Subject grouping

*S. no*		*Group age* *(years)*		*Sex*		*No. of subjects*	
1.		8-10		Male		20	
				Female		20	
2.		10-12		Male		20	
				Female		20	
3.		12-14		Male		20	
				Female		20	
4.		14-16		Male		20	
				Female		20	

### Criteria for selection

All the subjects were well-nourished and had no history of known serious illness, had undergone neither previous orthodontic treatment nor extraction of any permanent teeth, had normal dental conditions, had no previous history of trauma or injury to the face and the hand and wrist regions.

The patients and parental consent was taken before taking radiographs.

## METHODS

A total of 160 periapical radiographs of the mandibular right canines was taken by using bisecting angle technique with a standard size (31 × 41 mm Kodak) periapical dental X-ray film.

From several investigations, the tooth calcification of homologous teeth was found to be symmetrical; therefore, only right mandibular canine was examined. In the case of any missing right mandibular tooth the corresponding left mandibular tooth was substituted.

A total of 160 periapical radiographs of the MP3 region was taken by instructing the subject was to place the right hand with the palm downward in a flat table with the middle finger being centered on a 31 mm × 41 mm periapical dental X-ray film, parallel with the long axis of the film and the cone of the dental X-ray machine (70 kVp and 8 mA) positioned in slight contact with the middle phalanx, perpendicular to the film. Exposure time was 0.4 seconds.

All radiographs was processed with standardized processing technique and numbered for identification. Radiographs of high clarity and good contrast were used and any patients who presented congenital or acquired abnormalities of the phalanges were eliminated and interpretation of all radiographs were undertaken without referring to clinical data of age of patient.

Radiographic interpretation of this study was made as per the system developed to interpret skeletal and dental maturation.

♦ The development of the MP3 stages of the hand were evaluated using R.Rajagopal’s ^5^ observational scheme which is an modification of Hagg and Taranger’s observational scheme (Chart 1).
♦ The development of mandibular canine was assessed according to Demirjian’s stages ^6^ of dental calcification (Chart 2).

## CHART 1

**Modified MP3 Stages**

Stage 1: MP3-F Stage

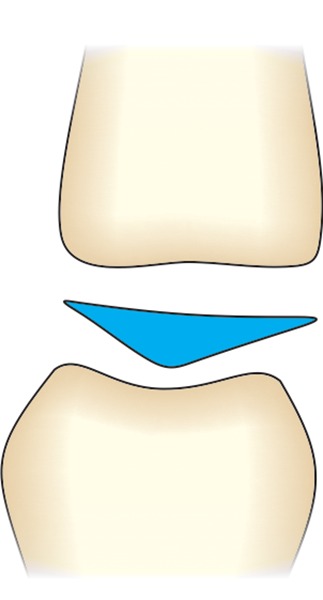



*MP3-F stage:* Start of the curve of pubertal growth spurt.

Epiphysis is as wide as metaphysis.

Ends of epiphysis are tapered and rounded.

Metaphysis shows no undulation.

Radiolucent gap between epiphysis and metaphysis is wide.

Stage 2: MP3-FG Stage

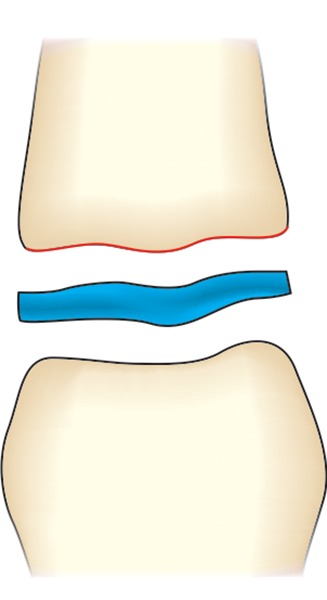



*MP3-FG stage:* Accleration of the curve of pubertal growth spurt.

Epiphysis is as wide as metaphysis.

Distinct medial and/or lateral border of epiphysis forms line of demarcation at right angle to distal border.

Metaphysis begin to show slight undulation.

Radiolucent gap between epiphysis and metaphysis is wide.

Stage 3: MP3-G Stage

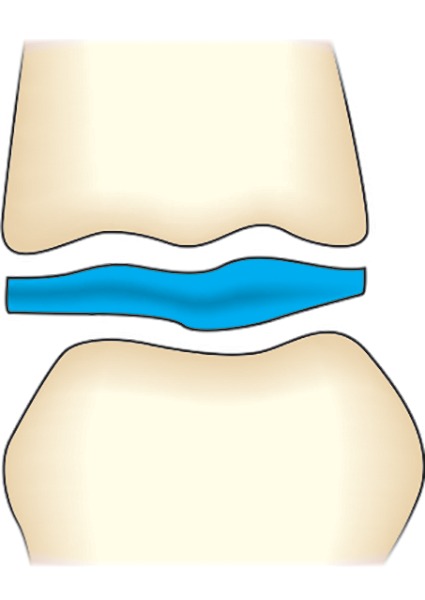



*MP3-G stage:* Maximum point of pubertal growth spurt Sides of epiphysis have thickened and cap its metaphysis, forming sharp distal edge one or both sides.

Marked undulations in metaphysis give it “Cupid’s bow” appearence.

Radiolucent gap between epiphysis and metaphysis is moderate.

Stage 4: MP3-H Stage

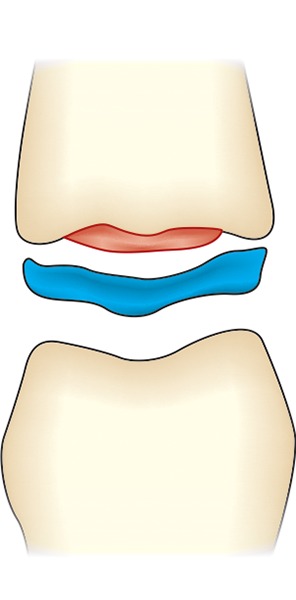



MP3-H stage: Deceleration of the curve of pubertal growth spurt.

Fusion of epiphysis and metaphysis begins.

One or both sides of epiphysis form obtuse angle to distal border.

Epiphysis is beginning to narrow.

Slight convexity is seen under central part of metaphysis. Typical “Cupid’s bow” appearance of metaphysis is absent, but slight undulation is distinctly present.

Radiolucent gap between epiphysis and metaphysis is narrower.

Stage 5: MP3-HI Stage

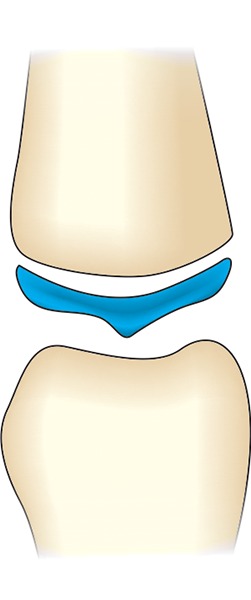



*MP3-HI stage:* Maturation of the curve of pubertal growth spurt.

Superior surface of epiphysis shows smooth concavity.

Metaphysis shows smooth, convex surface, almost fitting into reciprocal concavity of epiphysis.

No undulation is present in metaphysis.

Radiolucent gap between epiphysis and metaphysis is insignificant.

1. *Stage 6: MP3-1 Stage*

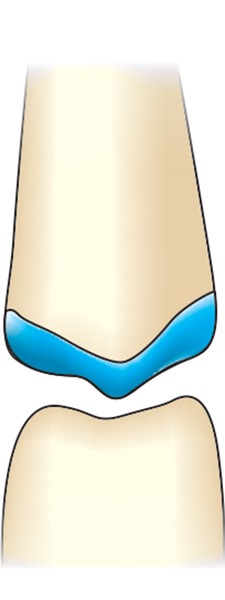



*MP3-I stage:* End of pubertal growth spurt.

Fusion of epiphysis and metaphysis is complete.

No radiolucent gap exists between metaphysis and epiphysis.

Dense, radiopaque epiphyseal line forms integral part of proximal portion of middle phalanx.

## CHART 2

**Table:** 

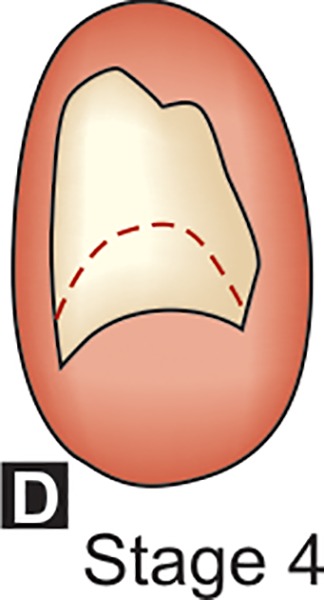		**D:** The crown formation is completed down to the CEJ. The superior border of the pulp chamber in the uniradicular teeth has a definite curved form, being concave towards the cervical region. The projection of the pulp horns, if present, gives an outline shaped like an umbrella top.	
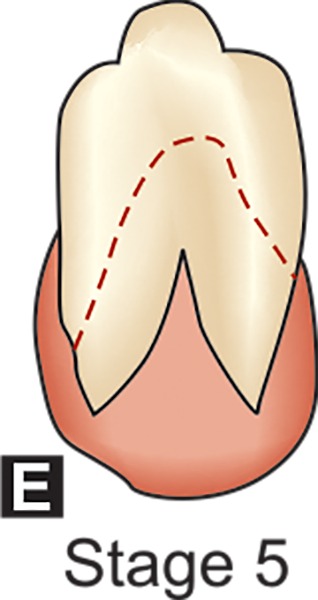		**E:** The walls of the pulp chamber now forms straight lines whose continuity is broken by the presence of the pulp horn which is larger than in the previous stage. The root length is less than crown height.	
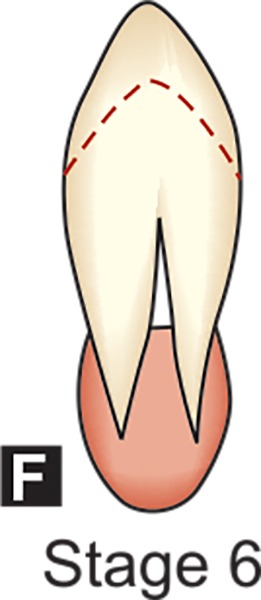		**F:** The walls of the pulp chamber now form a more or less isosceles triangle. The apex ends in a funnel shape. The root length is equal to or greater than the crown height.	
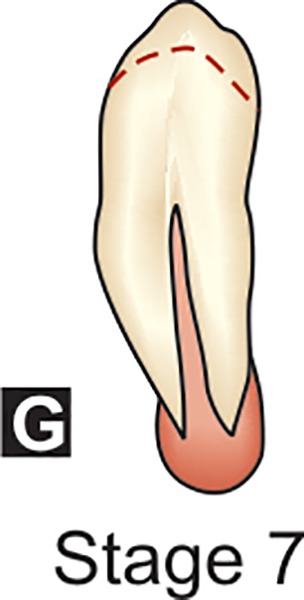		**G:** The walls of the root canal are now parallel and its apical end is still partially open.	
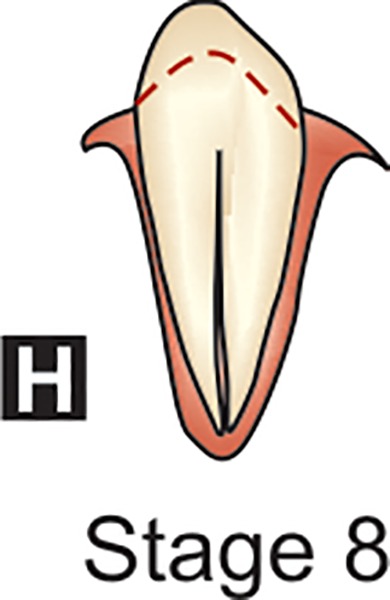		**H:** The apical end of the root canal is now completely closed. The periodontal membrane has a uniform width around the root and the apex.	

## DENTAL FORMATION STAGES D-H OF MANDIBULAR CANINE (DEMIRJAIN ET AL)

### Results

**Table 2:** **Table 2:** Shows the comparison of chronological age between male and female subjects in different groups using student’s unpaired ’t’ test

*Group*				*Sex*		*N*		*Mean*		*Std.* * Deviation*		*t*	
8-10		Age		Male		20		8.8750		0.42530.9930					
				Female		20		8.7250		0.5250		p = 0.327 ns	
10-12		Age		Male		20		10.7000		0.8176		0.1150	
				Female		20		10.7250		0.5250		p = 0.909 ns	
12-14		Age		Male		20		12.8000		0.4974		0.4480	
				Female		20		12.8750		0.5590		p = 0.657 ns	
14-16		Age		Male		20		14.8000		0.5231		0.2520	
				Female		20		14.7500		0.7164		p = 0.802 ns	

**Table 3:** **Table 3:** Shows the development stages of MP3 in male and female subjects using student’s unpaired ’t’ test

*Group*				*Sex*		*N*		*Mean*		*Std.* * Deviation*		*t*	
8-10		MP3		Male		20		1.7000		0.8013		2.8060	
				Female		20		2.4500		0.8870		p = 0.008 hs	
10-12		MP3		Male		20		2.3500		0.5871		4.3460	
				Female		20		3.2500		0.7164		p = 0.001 vhs	
12-14		MP3		Male		20		3.6500		1.0400		5.1310	
				Female		20		5.1000		0.7182		p = 0.001 vhs	
14-16		MP3		Male		20		5.4000		0.5026		2.7570	
				Female		20		5.8000		0.4104		p = 0.009 hs	

**Table 4:** **Table 4:** Shows development stages of mandibular right canine in male and female subjects using student’s unpaired ’t’ test

*Group*				*Sex*		*N*		*Mean*		*Std.* * Deviation*		*t*	
8-10		DEV		Male		20		4.4000		0.5026		2.1250	
				Female		20		4.7000		0.4702		p = 0.009 hs	
10-12		DEV		Male		20		5.4500		0.5104		0.6200	
				Female		20		5.5500		0.5104		p = 0.539 ns	
12-14		DEV		Male		20		6.3000		0.4702		3.1110	
				Female		20		6.7500		0.4443		p = 0.004 hs	
14-16		DEV		Male		20		7.3500		0.4894		2.3070	
				Female		20		7.7000		0.4702		p = 0.027 sig	

**Table 5:** **Table 5:** Correlations between MP3 stages and development stages of the mandibular canine-Males

*Group*		*Sex*						*MP3*	
8-10		Male		DEV		r		0.314	
						p		0.178 ns	
						N		20	
10-12		Male		DEV		r		0.501	
						p		0.025 sig	
						N		20	
12-14		Male		DEV		r		0.657	
						p		0.001 vhs	
						N		20	
14-16		Male		DEV		r		0.685	
						p		0.001 vhs	
						N		20	

**Table 6:** **Table 6:** Correlations between MP3 stages and development stages of the mandibular canine-Females

*Group*		*Sex*						*MP3*	
8-10		Female		DEV		r		0.593	
						p		0.006 hs	
						N		20	
10-12		Female		DEV		r		0.612	
						p		0.004 hs	
						N		20	
12-14		Female		DEV		r		0.742	
						p		0.001 vhs	
						N		20	
14-16		Female		DEV		r		0.764	
						p		0.001 vhs	
						N		20	

**Table 7:** **Table 7:** Correlations between MP3 stages and chronological age-Males

*Group*		*Sex*						*MP3*	
8-10		Male		MP3		r		0.116	
						p		0.626 ns	
						N		20	
10-12		Male		MP3		r		0.395	
						p		0.085 ns	
						N		20	
12-14		Male		MP3		r		0.570	
						p		0.009 hs	
						N		20	
14-16		Male		MP3		r		0.420	
						p		0.048 sig	
						N		20	

**Table 8:** **Table 8:** Correlations between MP3 stages and chronologica age-Females

*Group*		*Sex*						*MP3*	
8-10		Female		Age		r		0.449	
						p		0.047 sig	
						N		20	
10-12		Female		Age		r		0.342	
						p		0.083 ns	
						N		20	
12-14		Female		Age		r		0.623	
						p		0.003 hs	
						N		20	
14-16		Female		Age		r		0.337	

**Table 9:** **Table 9:** Correlations between developmental stages of man dibular canine and chronological age-Males

*Group*		*Sex*						*Dev*	
8-10		Male		Age		r		0.392	
						p		0.057 ns	
						N		20	
10-12		Male		Age		r		0.308	
						p		0.062 ns	
						N		20	
12-14		Male		Age		r		0.495	
						p		0.026 sig	
						N		20	
14-16		Male		Age		r		0.493	
						p		0.027 sig	
						N		20	

**Table 10:** **Table 10:** Correlations between developmental stages of mandibular canine and chronological age-Females

*Group*		*Sex*						*Dev*	
8-10		Female		Age		r		0.714	
						p		0.001 vhs	
						N		20	
10-12		Female		Age		r		0.392	
						p		0.068 ns	
						N		20	
12-14		Female		Age		r		0.927	
						p		0.001 vhs	
						N		20	
14-16		Female		Age		r		0.303	
						p		0.072 ns	
						N		20	

**Table 11:** **Table 11:** Gives the frequency distribution of mandibular canine calcification stages with MP3 stages-Males

*Canine* *calcification* *stages*		*MP3-F*		*MP3-FG*		*MP3-G*		*MP3-H*		*MP3-HI*		*MP3-I*		*Total*	
D		–		–		–		–		–		–		–	
E		9		1		–		–		–		–		10	
		90%		10%										100%	
F		16		5		1		–		–		–		22	
		72.7%		22.7%		4.6%								100%	
G		2		3		15		3		–		–		23	
		8.6%		13%		65.4%		13%						100%	
H		–		–		1		2		4		10		17	
				5.8%		11.7%		23.5%		59%		100%			

**Table 12:** **Table 12:** Gives the frequency distribution of mandibular canine calcification stages with MP3 stages-Females

*Canine* *calcification* *stages*		*MP3-F*		*MP3-FG*		*MP3-G*		*MP3-H*		*MP3-HI*		*MP3-I*		Total*	
D		–		–		–		–		–		–		–	
E		12		3				–		–		–		15	
		80%		20%										100%	
F		14		9		2		–		–		–		25	
		56%		36%		8%								100%	
G		–		1		14		6		2		–		23	
				4.6%		60.9%		26.1%		8.7%				100%	
H								1		3		11		15	
		–		–		–		6.6%		20%		73.4%%		100%	

* MP3 (F + FG + G + H + HI + I)

## DISCUSSION

Skeletal maturity assessment and growth prediction is an important tool in the interceptive orthodontic diagnosis and treatment planning.^7,8^ Chronological age is not a critical factor in the evaluation of the growth potential .^9^

Hagg and Taranger noted that the stages of ossification of middle phalynx of third finger of hand (MP3) follow the pubertal growth spurt from start to the end^10^ and R Rajagopal and Sudhanshu Kansal reported that recording modified MP3 stages using periapical X-ray film can be an accurate, simple, practical, and economical growth indicator for making decisions on treatment timing.^5^ Relationship between skeletal maturation and dental maturation is very poor according to Garn SM and Lewis AB,^11^ but Sierra AM,^12^ Chertkow S^3^ and Sandra Coutinho^4^ have shown strong correlation among dental development and onset of pubertal growth spurt and also said that there was racial variations.

Nolla CM^13^ and Demirjiam A,^6^ states that tooth mineralization stages are preferable to ages of tooth eruption in the assessment of dental maturity because mineralization is affected much less by local environmental influences and is measurable over a much longer period of time. Since the maturity indicators of brief duration are more informative than those of long duration, it may be advantageous to introduce new intermediate stages.

The purpose of this study was to determine whether the stages of calcification of the mandibular canine could be correlated with the six modified MP3 stages. It was also needed to know if this proposed method of using the developmental stages of the mandibular canine using an IOPA film and standard X-ray machine alone could be used as a single reliable factor in assessing the skeletal maturity.

The age group (8-16 years) selected in this study was on the basis of other maturation studies and as orthodontic treatment is frequently performed at this age group, skeletal age assessment becomes most critical.^14^

The radiographs of 160 healthy children were taken to assess the MP3 stage and development of mandibular right canine in the dental (IOPA) X-ray film.

In the present study radiographic assessment of stages of calcification based on Demirjian’s classification^6^ was used which has five different stages from (D-H). It is the most precise and simple method of assessment. Radiographic interpretation of the MP3 region was done using R Rajagopal et al^5^ (2002) modified MP3 stages.

On comparing the mean age of the male and female subjects in different groups as shown in Table 2, it is observed that they are not significant (p > 0.05) from each other. Insignificant differences in mean variation indicate that the sample used in this study is homogenous.

The results of the study reveal that the maturation of the middle phalanx of third finger, and the mandibular canine progress with advancing age. It can be seen from Tables 3 and 4 that there are gradually increasing stages of MP3 stages and canine development stages with age. The skeletal and dental maturation are progressing during the growth period but at a different rate. Studies by Hunter ^15^ have also reported similar findings.

It was found that at the same chronological age, there was highly significant difference (p < 0.01) in each group (Table 3). In all the groups MP3 stages were more advanced in females than in males. Similar types of sexual dimorphism regarding the maturational parameters have been earlier reported by Hunter (1966),^15^ and Hagg and Taranger^10^ (1982).

On comparing the developmental stages of mandibular right canine in male and female groups, it showed that there was a very highly significant difference in all the groups except in the group (10-12 years) where it was not significant. This indicates an inconsistent sexual dimorphism (Table 4). Studies by Chertkow^3^ (1979) also support this results.

The correlation coefficient was studied between MP3 stages and developmental stages of mandibular right canine (Tables 5 and 6) using Karl Pearsons test. In males, the correlation was not significant (p > 0.05) in the group (8-10 years), in the group (10-12 years) it was significant (p < 0.05) and in the group (12-14 years) and (14-16 years) it was very highly significant (p < 0.001).

In females, the correlation coefficient was highly significant (p < 0.01) in the group (8-10 years) and (10-12 years) and it was very highly significant (p < 0.001) in the groups (12-14 years) and (14-16 years).

The correlation coefficient was studied between MP3 stages and chronological age (Tables 7 and 8) using Karl Pearsons test. In males, the correlation was found to be not significant (p > 0.05) in the group (8-10 years) and (10-12 years), it was highly significant (p < 0.01) and in the group (12-14 years) it was significant (p < 0.05).

In females, the correlation was found to be significant (p < 0.05) in the group (8-10 years), it was not significant (p < 0.05) in the group (10-12 years) and (14-16 years), whereas in the group (12-14 years) it was highly significant (p < 0.01).

On comparing the developmental stages of mandibular right canine and the chronological age in males and females (Tables 9 and 10) using Karl Pearsons test, in males the correlation was found to be not significant (p > 0.05) in the group (8-10 years) and (10-12 years), it was highly significant (p < 0.01) in the group (12-14 years) and significant in the group (14-16 years).

In females, correlation was found to be very highly significant (p < 0.001) in the group (8-10 years) and (12-14 years), it was not significant in the group (10-12 years) and (14-16 years).

This indicates that there is an inconsistent correlation between chronological age and dental maturation. Similar findings were reported by Demirjian A,^6^ Buschang PH.^4^

The association between mandibular canine development and MP3 stages also allows the clinician to more easily identify the early stages of the pubertal growth spurt. By using United States reference data for comparison,^4^ the initiation of the spurt is indicated by canine stage F. Stage G, which coincides with the eruption of the canine into the oral cavity, occurs approximately one year before the PHV in boys, but occurs 5 months before the PHV in girls. This may reflect hormonal changes which accompany puberty. The relationship between calcification of the mandibular canine and MP3 stages were quite high when analyzed statistically.

For males (Table 11), the D stage of canine calcification had no subjects from the samples, the E and F stages showed the maximum correlation with the MP3-F stage (90% and 72.7%, respectively), while in the G stage a high correlation was seen with the MP3-G stage (65.4%). In the H stage of mandibular canine calcification, the MP3-I stage was observed to have a high correlation (59%). For females (Table 12), the correlations were closely tallying as those of the males. Here again, the D stage of mandibular canine calcification had no subjects from the samples, the E and F stages showed maximum correlation with the MP3-F stage (80% and 56%, respectively). The G stage seemed to show a high correlation with the MP3-G stage (60.9%) and the H stage closely related with the MP3-I stage (73.4%).

These figures indicate that in both males and females, the D stage of canine calcification had no subjects in the MP3 stages, while the remaining stages of canine calcification showed high correlations with the MP3 stages in both genders.

## CONCLUSION

From this study the following conclusions can be drawn:

1. The correlation between canine calcification stages and MP3 stages was found to be of high statistical significance.
2. The developmental stages of the mandibular canine could be used very reliably as a sole indicator in assessing the skeletal maturity.
  – The stage E and stage F of mandibular canine calcification coincided with the MP3-F stage indicating 80-100% of pubertal growth remaining.
  – The stage G of mandibular canine calcification coincided with the MP3-G stage indicating 25-65% of pubertal growth remaining.
  – The stage H of mandibular canine calcification coincided with the MP3-I stage indicating completion of pubertal growth spurt.
3. The proposed method of assessing the skeletal maturity using a standard sized IOPA film and a standard dental X-ray machine was found to be simple and practical.

## References

[1] García-Fernandez P, Torre H, Flores L, Rea J (1998). The cervical vertebrae as maturational indicators.. J Clin Orthod.

[2] Abdel-Kader HM (1998). The reliability of dental X-ray film in assessment of MP3 stages of the pubertal growth spurt.. Am J Orthod Dentofacial Orthop.

[3] Chertkow S (1980). Tooth mineralization as an indicator of the pubertal growth spurt.. Am J Orthod.

[4] Coutinho SH, Buschang PH, Miranda F (1993). Relationships between mandibular canine calcification stages and skeletal maturity.. Am J Orthod Dentofacial Orthop.

[5] Rajagopal R, Kansal S (2002). A comparison of modified MP3 stages and the cervical vertebrae as growth indicators.. J Clin Orthod.

[6] Demirjian A, Levesque GY (1980). Sexual differences in Dental develop-ment and prediction of emergence.. J Dent Res.

[7] Pancherz H, Hagg U (1985). Dentofacial orthopedics in relation to somatic maturation. An analysis of 70 consecutive cases treated with Herbst appliance.. Am J Orthod.

[8] Kopecky GR, Fishman LS (1993). Timing of cervical headgear treatment based on skeletal maturation.. Am J Orthod Dentofacial Orthop.

[9] Kucukkeles N, Acar A, Biren S, Arun T (1999). Comparisons between cervical vertebrae and hand-wrist maturation for the assessment of skeletal maturity.. J Clin Peadiatr Dent.

[10] Hagg U, Taranger J (1982). Maturation indicator and the pubertal growth spurt.. Am J Orthod.

[11] Garn SM, Lewis AB, Bonne B (1962). Third molar formation and its development course.. Angle Orthod.

[12] Sierra AM (1987). Assessment of dental and skeletal maturity: A new approach.. Angle Orthod.

[13] Nolla CM (1960). The development of the permanent teeth.. J Dent Child.

[14] Negi KS, Sharma VP, Kapoor DN, Tandon P (2003). Assessment of growth impetus using MP3 maturation and its correlation with CVMI and dental age.. J Indian Orthod Soc.

[15] Hunter CJ (1966). The correlation of facial growth with body height and skeletal maturation at adolescence.. Angle Orthod.

